# Research on Live Working Robots for 10 KV Distribution Networks Adopting Four-Dimensional Safety Guarantee Framework

**DOI:** 10.3390/s26144535

**Published:** 2026-07-17

**Authors:** Xiaohui Xie, Lining Sun, Pan Luo, Xiang Yin

**Affiliations:** 1 Robot and Microsystem Technology Research Center, Soochow University, Suzhou 215137, China; 2 Robotic Research Institute, Harbin Institute of Technology, Harbin 150001, China

**Keywords:** 10 kV distribution network, live working robot, tracked insulated spider aerial vehicle, dual 6-DOF insulated manipulators, multi-modal perception, 13-DOF cooperative control, four-dimensional safety guarantee framework

## Abstract

Traditional manual 10 kV live-line maintenance is accompanied by high personal risks and incomplete safety protection, while overall operational efficiency is limited. This paper develops an intelligent live-working robot based on a tracked insulated spider aerial vehicle. The system is equipped with vertical lifting modules and a pair of 6-DOF insulated manipulators to form a 13-DOF integrated motion platform. Binocular cameras, LiDAR, real-time insulation monitors, and electromagnetic interference detectors are integrated as multi-modal sensing hardware to achieve high-precision positioning of overhead lines and pole fittings. A master–slave collaborative control strategy combined with mixed reality (MR) and visual auxiliary force feedback is proposed to coordinate the tracked chassis, lifting structure, and dual manipulators. A four-dimensional full-cycle safety guarantee framework is further constructed, covering insulation protection, anti-interference communication, human–machine risk avoidance, and full-task supervision to support real-time early warning and motion interlock. Field tests on actual 10 kV distribution lines verify stable positioning performance under controlled test conditions, and no safety accidents occurred in all trials. The designed robotic system provides an optional technical scheme for intelligent unmanned live-line maintenance of distribution networks.

## 1. Introduction

### 1.1. Domestic and Foreign Research Progress

The 10 kV distribution network acts as the terminal core of China’s power supply system, and live-line maintenance guarantees uninterrupted power supply. Conventional manual live tasks expose operators to electric shock, arc, and electromagnetic radiation hazards [1,2].

In recent decades, research teams in Japan, Canada, China, and other countries have developed power operation robots and completed preliminary field trials [3,4,5]. Compared with inspection robots, live-working manipulator robots progress more slowly, and most prototypes can only carry out simple maintenance operations. Diverse on-site tasks and customized functional requirements bring clear challenges to prototype development.

Research on distribution network live-line working robots can be divided into three developmental phases, starting from the late 20th century. The first generation, represented by Japan’s Phase II robot, lacked auxiliary visual sensors, and operators relied on direct observation and panel input to complete operations [3]. Second-generation equipment adopted master–slave structures with positioning sensors, allowing ground operators to control dual manipulators via heterogeneous master arms and keyboards [4]. Third-generation robots generally use 6-DOF hydraulic manipulators, solving the overweight and insufficient load limits of second-generation prototypes [5,6].

All three generations rely on remote master–slave manipulation. Even with visual sensors, these devices only support real-time monitoring rather than full autonomous operation. Restricted by voltage adaptability and visual stability, most research remains at the laboratory testing stage. Only a small number of products, such as Japan’s Phase III robot, have been formally deployed, and manual maintenance still dominates global live-line work.

China features vast territory, uneven energy allocation, and huge power demand in coastal load areas, creating continuous demand for power equipment inspection and maintenance. Joint research between power enterprises, research institutes, and manufacturers accelerates the iteration of domestic power robots.

Domestic research on live-working robots started later than foreign studies, yet yielded abundant research outputs. The earliest domestic prototype was jointly developed by Shandong Electric Power Research Institute and Shandong Luneng Intelligent Technology Co., Ltd. (Jinan, China) in 1999 [7]. Operators controlled dual manipulators inside insulated buckets, but the bulky structure only supported basic playback without practical engineering value. Current third-generation domestic robots integrate multiple sensors for remote control, and can complete wire connection, insulator replacement, deicing, and foreign object removal [8].

In 2018, Tsinghua University Tianjin Advanced Equipment Research Institute designed dedicated wire stripping clamps and wire fixtures matching 10 kV live robots with acceptable on-site effects [9]. In 2019, State Grid Shanghai Electric Power Co., Ltd. (Shanghai, China) launched an outdoor robot equipped with LiDAR and industrial cameras for environmental reconstruction, autonomous navigation, and path planning [10]. In the same year, State Grid Tianjin Electric Power Co., Ltd. (Tianjin, China) and NARI Group co-developed the “Dawn” robot with optimized visual recognition, motion control, and anti-electromagnetic-interference performance [11]. In 2020, State Grid Hunan Electric Power Co., Ltd. (Changsha, China) and Wuhan Textile University designed matched auxiliary tools, including wire clamps, stripping devices, and cable cutters, and finished prototype manufacturing under remote control mode [12].

Two typical characteristics exist in the perception environment of live-working robots. On one hand, operation scenes are relatively static, reducing temporary perception failures. LiDAR and cameras achieve stable close-range detection under various weather conditions and become mainstream sensing equipment. As early as the 1980s, the Phase robot jointly developed by Kyushu Electric Power and Yaskawa Electric carried laser ranging and stereo vision cameras, which have remained core sensing hardware ever since [4].

On the other hand, pole-mounted fittings are vertically distributed, while mainstream cameras and LiDAR feature wide horizontal but narrow vertical fields of view. Single-frame sensor data cannot complete full scene reconstruction, and vertical sensor alignment requires manual adjustment unavailable in field tasks. Single-frame LiDAR point clouds are sparse, making thin overhead conductors hard to detect accurately. Multi-sensor fusion thus becomes the mainstream perception solution for distribution network scenes [6,13].

Although China has realized independent development of semi-automatic live-working robots, most prototypes stay in laboratory verification stages. Large-scale promotion faces two core bottlenecks: safety guarantee and operation efficiency.

From the safety perspective, existing equipment lacks mature solutions for operation error interlock and integrated insulation testing. Most insulated vehicles adopt single-arm structures without coordinated control of chassis, lifting platforms, and manipulators. Severe electromagnetic interference, strict insulation standards, and narrow working spaces further hinder stable high-precision operation [14,15,16]. This paper constructs a four-dimensional safety constraint framework covering insulation, anti-interference communication, human–machine risk prevention, and full-process monitoring to realize redundant on-site protection.

From the efficiency perspective, two main constraints exist. First, live tasks require various special tools with inconsistent mechanical interfaces. Frequent tool replacement and complex procedures lower overall efficiency, highlighting the demand for universal intelligent end-effectors. Second, most perception algorithms assume the vehicle is manually adjusted to an ideal position before data collection. In field tests, operators spend much time adjusting vehicle positions via remote screens before formal sensing and operation.

To solve positioning delays, this paper builds a multi-sensor acquisition platform combining laser SLAM and binocular vision. Multi-sensor fusion calculates target coordinates and continuously reconstructs scenes during platform lifting. The system acquires the global position of the insulated vehicle and realizes autonomous positioning.

Most existing prototypes only rely on passive mechanical insulation without standardized third-party high-voltage and EMC testing for systematic safety assessment. Traditional teleoperation only provides 2D visual feedback without real-time force perception, and few papers analyze how operator experience affects task performance. To fill these gaps, this paper proposes a multi-sensor fusion perception framework, a quantitative four-dimensional safety constraint system, and an MR human-in-the-loop impedance control strategy.

### 1.2. Existing Technical Challenges and Main Contributions

This paper takes a 20-DOF live-working robot built on a crawler insulated spider boom vehicle as the test carrier, equipped with lifting modules and dual 6-DOF insulated manipulators. Hardware integration only serves as test hardware; core research focuses on perception frameworks, safety evaluation systems and human–machine control strategies. Table 1 compares traditional schemes and the proposed system, with three core contributions summarized below:

1. A vertical multi-frame point cloud fusion perception mode is proposed. Different from mainstream fixed-point data collection, this scheme continuously collects sensing information during the full lifting stroke to complete scene stitching reconstruction.

2. A four-dimensional safety constraint evaluation framework for multi-DOF linkage motion is constructed. Unlike passive insulation or single-index risk judgment, this system sets two response grades: early warning and motion locking across four monitoring dimensions.

3. An MR human-in-the-loop impedance coordination control mode is designed. Unlike fixed-gain traditional master–slave control, this scheme divides multi-level operation permissions and matched motion limits for different task stages.

A complete 20-DOF electromechanical platform integrating a crawler chassis, lifting slide rails, and dual manipulators is manufactured for experimental verification. Conventional live-working robots follow the pipeline of environment modeling, target recognition, and path planning. Diversified operation objects, narrow working spaces and field interference easily cause grasping deviation and clamping failure, requiring repeated manual fine-tuning and extending task duration [17,18,19].

As summarized in Table 1, the proposed framework supplements existing research at the perception, risk assessment, and cooperative control layers. Under four-dimensional safety constraints, the whole task flow is reorganized to fit field demands. The force-feedback MR teleoperation architecture avoids direct staff exposure to high-voltage environments and simplifies manual steps compared with traditional remote-control schemes.

## 2. System Design of Live Working Robot System

### 2.1. Operation Route and System Design

**Figure 1:**
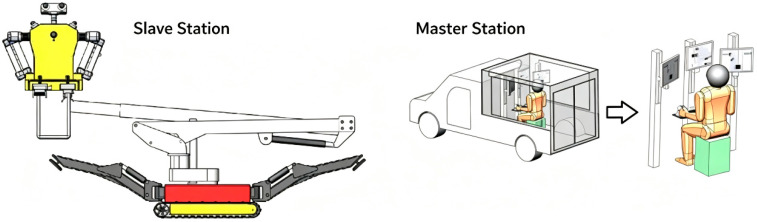
Effect diagram of the master–slave station of the power-line operation robot.

Figure 1 displays the overall rendering of master–slave robot stations. Targeting typical 10 kV live tasks including insulation peeling, conductor cleaning, and drainage wire locking, this paper develops a heterogeneous master–slave platform matching the proposed force-feedback teleoperation and four-dimensional safety framework. Virtual prototype simulation optimizes mechanical parameters to provide kinematic support for subsequent algorithm verification. As shown in Figure 1, the overall layered architecture consists of an upper master control terminal and a field slave execution subsystem.

**Figure 2:**
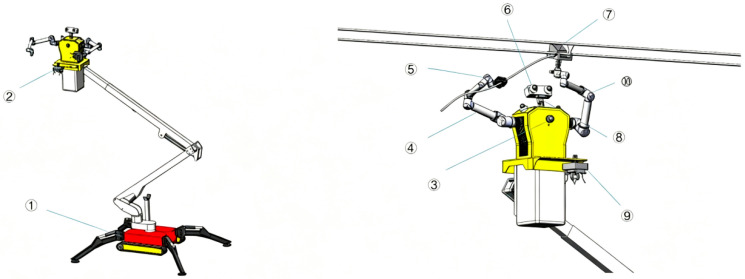
Process diagram of three-phase sparking for a live work robot in a distribution network. ① Spider boom vehicle; ② slave robot system; ③ lidar; ④ robotic arm 1; ⑤ clamp jaw; ⑥ panoramic camera; ⑦ wiring tool; ⑧ binocular camera; ⑨ wire stripping tool; ⑩ robotic arm 2.

Figure 2 presents standardized task workflows matched with dual-arm coordinated motion planning under multi-layer safety constraints. The slave robot integrates LiDAR, panoramic, and binocular vision to reduce wire swing interference induced by wind loads. Guided by hand-eye calibration algorithms, the left manipulator clamps wires to stabilize targets for precise insulation stripping conducted by the right manipulator. After peeling, the system automatically switches end-effectors to polish conductor impurities, then completes conductive grease coating and drainage wire installation via a coordinated dual-arm motion with automatic tool replacement. The slave robot is mounted on a tracked insulated spider chassis with telescopic outriggers to adapt to uneven rural terrain. A multi-joint rotatable boom realizes obstacle avoidance around poles and trees, while an independent vertical lifting unit carries two 6-DOF insulated manipulators.

The slave side adopts a 20-DOF redundant layout: 7 DOF for boom adjustment, 1 DOF vertical lifting platform, and two 6-DOF manipulators. Compared with single-arm robots, redundant DOFs expand feasible working space and pose adjustment diversity, providing sufficient kinematic redundancy for safety constraint solving.

### 2.2. Aerial–Ground Collaborative Live Working Robot System

**Figure 3:**
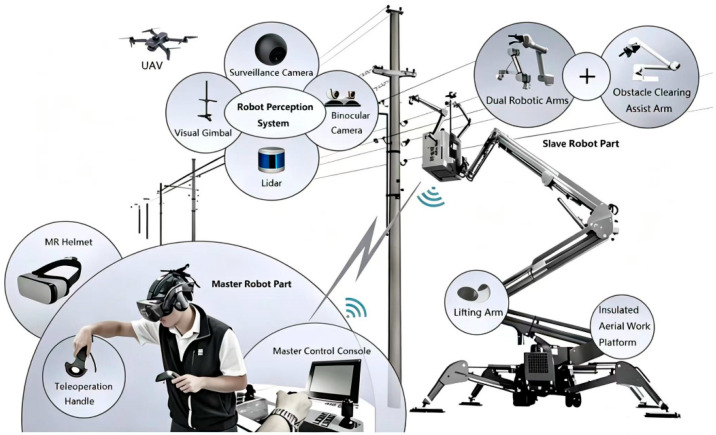
Overall architecture of aerial–ground collaborative live working robot system.

This paper constructs an aerial–ground integrated teleoperation architecture with local autonomous judgment logic, divided into four functional modules supporting the full workflow of the four-dimensional safety monitoring system (Figure 3): MR master control terminal, ground dual-arm execution platform, multi-modal perception fusion module, and auxiliary monitoring UAV.

1. Master station module: Twin force-feedback master manipulators realize bidirectional transmission of control instructions and contact force signals. MR head-mounted displays fuse multi-sensor scene data to reconstruct on-site environments and build human-in-the-loop conditions for real-time safety risk judgment.

2. Ground slave execution station: A tracked insulated vehicle equipped with interchangeable 6-DOF manipulator end-effectors adapts to rugged mountain terrain, acting as the hardware actuator for motion commands generated by algorithms.

3. Multi-modal perception module: LiDAR and binocular cameras extract wire features and detect surrounding obstacles. Fused sensing data serve as input for safety assessment and motion planning algorithms.

4. UAV aerial auxiliary monitoring: Drones provide top-down global perspectives for pre-task risk inspection and full-process task recording, forming double-layer supervision together with ground perception sensors.

**Figure 4:**
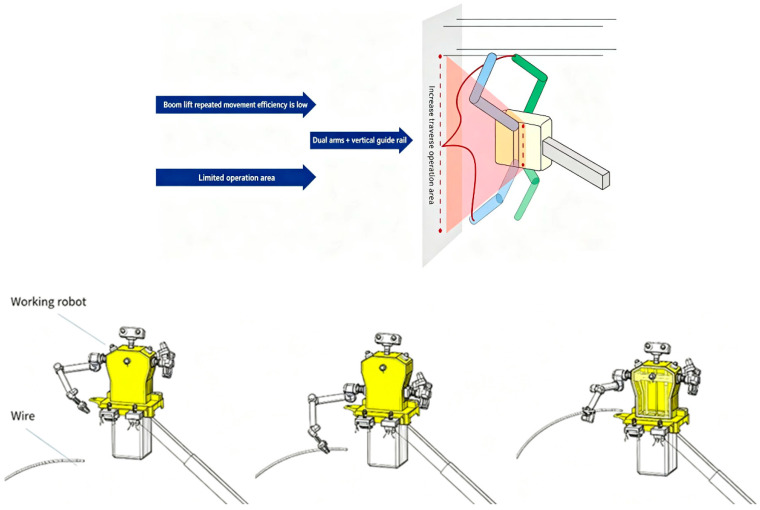
Dual-arm robot structure for increasing vertical workspace. Notes: The long red dashed line marks the expanded vertical operation range. The short red dashed lines represent the vertical lifting mechanism. The red shaded area denotes the effective vertical workspace covered by the dual-arm structure. The blue and green structures are dual auxiliary manipulators for workspace expansion, and the yellow parts are the unified robot main body without extra illustration.

Conventional lifting bucket vehicles suffer from a narrow vertical working stroke, requiring repeated position calibration by operators to satisfy three-phase wiring requirements. To tackle this problem, an independent lead-screw-driven lifting rail structure is designed in Figure 4. Each manipulator is fixed on a separate lifting slider with a single stroke of 1.3 m, and the superposed vertical operating range reaches over 2.6 m to satisfy one-time three-phase connection demands. Vision positioning results are fed back into the closed-loop control loop to adjust arm height automatically under safety constraints, reducing frequent manual calibration and boosting overall autonomy.

### 2.3. Four-Dimensional Safety System

To address the hidden hazards in 10 kV overhead line field tasks, this paper constructs a four-dimensional hierarchical safety evaluation framework, which systematically covers insulation physical barrier, electromagnetic communication reliability, human–robot interactive manipulation constraint, and whole-task procedure supervision. Different from conventional schemes that only adopt passive structural insulation or isolated single-index protection, this framework sets unified monitoring indicators and two-tier response rules (early warning and motion locking) for four independent dimensions, forming a standardized whole-process risk supervision architecture for master–slave live-line operation robots.

#### 2.3.1. Insulation Body Safety

Insulation performance forms the fundamental high-voltage physical barrier. Complex distribution scenes carry breakdown and flashover risks if insulation fails. Matching human–machine teleoperation modes, the robot adopts a layered multi-stage insulation scheme with graded creepage distance and isolation standards rather than single insulating materials. Four layered isolation measures are defined:

1. Insulating connectors are arranged between the end effector and the manipulator arm to isolate potential voltage conduction paths and extend the effective creepage distance;

2. The outer surface of the operation end tools is coated with dedicated insulating paint for local isolation protection;

3. Full wrapping insulating sleeves are installed on each manipulator joint to prevent phase-to-phase or ground short-circuit caused by accidental contact with irrelevant conductors;

4. The whole vehicle platform and lifting bucket mechanism are equipped with bottom insulating components to achieve isolation from ground potential.

All manipulator insulating accessories follow unified standards. Joint overlapping sections maintain continuous isolation, supporting 20 kV withstand voltage for 3 min with flashover and breakdown thresholds above 30 kV. Real-time modules continuously monitor insulation resistance, surface temperature, and structural integrity during operation. The slave prototype completed third-party high-voltage testing per domestic standards: under 45 kV AC voltage lasting 1 min, no breakdown, flashover or overheating occurs, with leakage current controlled below 100 μA. Test results verify that the layered insulation structure meets long-term 10 kV live-line operation demands under laboratory conditions.

#### 2.3.2. Electromagnetic Communication Safety

Stable data interaction between the master ground station and field slave robot constitutes the second core dimension of the safety framework. Long-distance transmission of video streams, force feedback data, and control commands faces ambient power-frequency electromagnetic interference under overhead lines. This system selects WiFi6 wireless communication modules to support dual 2.4 GHz/5 GHz frequency bands, guaranteeing an effective operation communication distance of over 50 m. Since 10 kV power lines only radiate 50 Hz fundamental frequency and low-order harmonics below 3 kHz, targeted EMC layout measures replace full overall shielding, including power supply filtering circuits, partial module shielding, standardized grounding wiring, and routing isolation away from high-current switching branches.

Fixed hardware encoding and frequency division transmission logic are configured to isolate video streaming and motion control signals. Video data adopts GPU compression coding for image transmission, while control instructions occupy an independent frequency channel to reduce mutual bandwidth occupation. A fixed periodic handshake mechanism is formulated as the judgment criterion for communication link health status: the master station transmits handshake packets every 2.5 s. If the slave side receives no valid handshake signal within 2.8 s, the system judges the link to be abnormal. The slave manipulator will immediately suspend all executing actions and stay in standby locking state until the communication handshake recovers.

Complete EMC immunity tests (electrostatic discharge, alternating magnetic field immunity) are conducted in qualified laboratories referring to GB/T 17626.2 series standards [20]. Test records prove that the communication module can maintain continuous data interaction without signal interruption under preset interference intensity, which serves as the verification basis for this communication safety dimension.

#### 2.3.3. Human–Robot Interaction Safety

This dimension focuses on risk restriction during remote human manipulation, relying on multi-sensor environment collection and master–slave motion logic judgment to avoid collision triggered by operator misoperation. LiDAR ranging equipment, visible-light and infrared cameras collect surrounding environmental information, and multi-source data fusion is realized under the ROS framework to reconstruct the surrounding obstacle distribution. The collected environmental information provides a boundary reference for subsequent motion constraint judgment.

A complete upper computer human–machine interaction interface is developed to display a visualized environmental model and operating state parameters for operators. On the master control terminal, the kinematic solution is executed for issued motion commands in advance. Combined with real-time obstacle spatial data, the system pre-checks whether the planned trajectory will trigger collision risks before forwarding commands to the execution manipulator, adding a pre-screening link to restrict improper operation actions.

**Figure 5:**
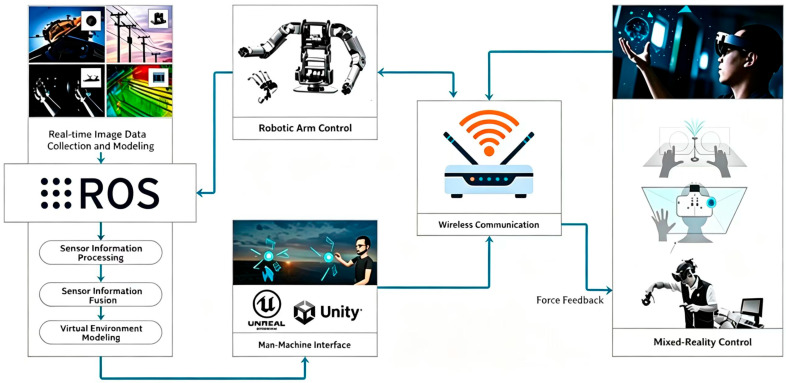
Interaction safety scheme of a human–robot system.

Time delay of force feedback signal transmission and presentation is a key factor affecting manipulation safety. However, there is a significant lag in the reproduction and transmission of force feedback to human hands, which is generally believed to be in the hundreds of milliseconds or even seconds. This kind of delay and lag is fatal to the safety of the operation, so it must be avoided [21,22,23]. To alleviate hidden risks brought by signal transmission lag, this framework designs a fixed display mode for force data. After force sensing data is uploaded to the master station, numerical results are presented via a real-time progress bar inside the MR helmet viewport, allowing operators to obtain intuitive force conditions synchronously and lower judgment errors caused by feedback delay, as demonstrated in Figure 5.

#### 2.3.4. Power-Line Operation Process Safety

This dimension introduces UAV auxiliary patrol to build a pre-operation inspection, mid-task monitoring, and post-operation acceptance three-stage supervision procedure, forming full-process procedural constraints to standardize operation steps and eliminate blind monitoring areas.

Prior to robot task startup, the UAV performs field environment reconnaissance to check meteorological indices, including ambient temperature (−10 °C–50 °C), relative humidity (below 95%), and on-site wind speed, complying with the environmental requirements specified in GB/T 18857 [24]. Meanwhile, aerial shooting is implemented to confirm the overall layout of fittings and lines, judging whether the scene satisfies the precondition for robot live-line work. During ongoing manipulation, the UAV adjusts its hovering perspective according to different working procedures. It feeds back the relative position between manipulator arms and adjacent non-working-phase lines in real time, and uses infrared temperature detection to monitor temperature variation in key metal connecting components, providing an auxiliary reference for ground operators. After all operation actions finish, the UAV carries out a post-inspection patrol: infrared thermal scanning is used to check the temperature of newly installed wire clamps and inspect the fastening state of bolts and assembly completeness of accessories. After all items pass inspection, aerial photos are archived to complete closed-loop task recording. The introduction of aerial auxiliary equipment realizes multi-angle procedural supervision as a complementary part of the four-dimensional safety architecture.

## 3. Implementation Schemes for Safety and Efficiency

### 3.1. Mixed Reality Environment with Vision-Force Haptic Fusion Feedback

**Figure 6:**
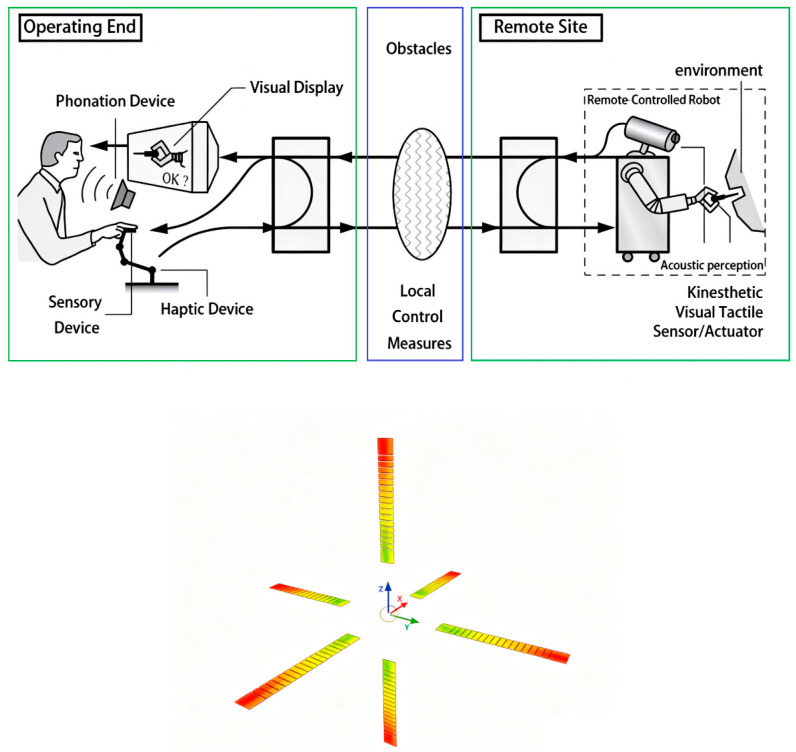
Multi-modal control and graphic force feedback illustration. Notes: The three-dimensional coordinate system at the center represents the spatial coordinate frame of the manipulator. The color transitions from green to yellow and then to red along each axis indicate the gradual growth of the feedback force value, where green stands for low force, yellow for medium force, and red for maximum force within the working range.

Figure 6 demonstrates the closed-loop control architecture constructed for this master–slave live working robotic system. Operators manipulate the slave manipulator through the master control terminal, and acquire field operating states relying on coupled visual feedback and tactile feedback to build immersive teleoperation observation conditions.

Most existing teleoperation systems face drawbacks in force information presentation, where lag mainly originates from the rendering display link rather than wireless data transmission. A delay of roughly 200 ms in force value presentation will hinder operators from perceiving contact collision in time, easily causing unintended mechanical impact during live-line manipulation. Targeting this operational hidden trouble, this paper designs an image-aided auxiliary force display framework matched with mixed reality panoramic rendering, rather than improving the underlying transmission filtering algorithm itself.

This auxiliary display structure acts as a supplement to physical hardware force feedback. Three-axis contact force magnitude is visualized inside the MR field of view by independent progress bars corresponding to X, Y, and Z directions. A fixed RGB color grading rule is formulated for state distinction: green corresponds to light contact load, blue represents medium contact force, and red denotes heavy contact extrusion. Variation in brightness within each color interval further reflects tiny fluctuations of contact force magnitude, as illustrated in the subgraph of Figure 6.

Common mainstream schemes adopt fixed UI windows fixed at the edge of the MR field of view. Such static windows will block a partial field of view and force operators to frequently shift their sight between operating targets and force indicator panels. This paper designs an end-effector-tracking embedded display structure; the force display bars follow the working end synchronously along with manipulator movement. This customized layout avoids sheltering charged fittings, reduces the frequent sight-switching burden for operators, and forms a differentiated human–computer observation layout compared with static panel display modes.

### 3.2. Multi-Modal Perception for Insulated Bucket Positioning

#### 3.2.1. Multi-Modal Perception: Sensor Selection and Implementation Methods

Targets distributed in 10 kV distribution operation scenarios can be divided into large-scale infrastructure, including overhead lines and poles, as well as small pole-mounted accessories such as insulators, drop fuses and wiring fittings. Different categories of objects require respective modal data for feature extraction. LiDAR is capable of generating precise depth point cloud data suitable for outlining large-scale frameworks; yet tiny accessories lose abundant geometric features in sparse point clouds, while binocular images retain complete texture appearance features for small components identification.

**Figure 7:**
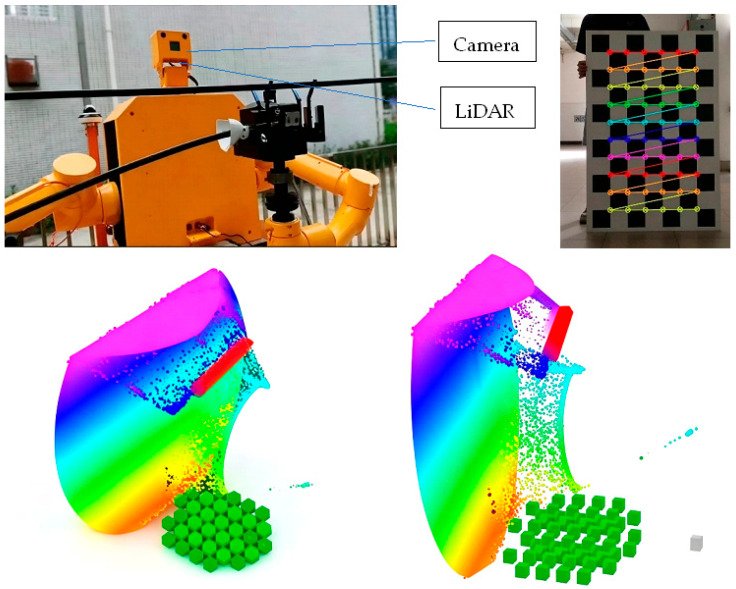
Perception: hardware, calibration, and mechanism. Notes: Top-left: Camera-LiDAR integrated hardware; Top-right: Calibration checkerboard. Bottom two: Raw and segmented power scene point clouds. Rainbow colors indicate depth; red = power lines, green = feasible parking area.

On this basis, this paper adopts the combined sensing configuration of Livox Mid70 solid-state LiDAR (Livox Technology, Shenzhen, China) plus ZED 2i binocular camera (Stereolabs, San Francisco, CA, USA), shown in the upper left area of Figure 7. The core design highlight lies in building a complete fixed calibration and data processing pipeline adapted to vertically arranged pole fittings, instead of proposing improved fusion algorithms. The whole workflow is designed to collect complementary depth and texture information to support subsequent target extraction and vehicle position calculation.

Accurate positioning of the insulated lifting bucket is a necessary prerequisite before starting clamping and wiring tasks. The traditional field operation mode requires workers to adjust the bucket parking position repeatedly by trial and observation, which consumes much preparation time and brings potential collision risks. Although the lifting slide rail on the robot expands the movable range of manipulators after the bucket is parked, an improper initial parking position still leads to repeated vehicle relocation. Hence, this dual-sensor matching pipeline is constructed to finish target recognition and solve the reasonable vehicle standby position. The following overall working procedure is displayed in Figure 7:

1. Complete extrinsic parameter calibration between LiDAR and binocular camera to solve the unified coordinate transformation matrix.

2. Realize coordinate system calibration between LiDAR and gimbal platform with standard 3D calibration targets.

3. Acquire kinematic mapping relations between gimbal and robot base through assembly drawing parameters plus auxiliary calibration tests.

4. Conduct conventional preprocessing for raw data: point cloud filtering and resampling, image distortion correction, and feature matching.

5. Adopt mature Kalman filtering for cross-modal data fusion. Since the optimized fusion algorithm is not the research focus of this paper, relevant mathematical derivation formulas are omitted here.

6. Implement instance segmentation on RGB images to screen wire targets, then map segmented results into point cloud coordinate space to accomplish 3D target segmentation. Binocular visual boundary detection results are projected onto a wide-range point cloud to calculate a reasonable parking area for the whole vehicle.

The subgraphs in Figure 7 present processing outcomes: original point cloud scene, segmented overhead power lines marked in red, LiDAR origin coordinate in gray, and the solved feasible parking zone framed by green bounding cubes. This dual-sensor collocation makes use of the long-distance depth detection advantage of LiDAR and the high texture recognition capability of binocular vision. The designed fixed calibration and data processing pipeline forms a complete working flow for this vertical tower scene, providing a standardized process to reduce blind repeated vehicle relocation in practical field work.

#### 3.2.2. Accurate Moving Algorithm of Insulated Bucket

**Figure 8:**
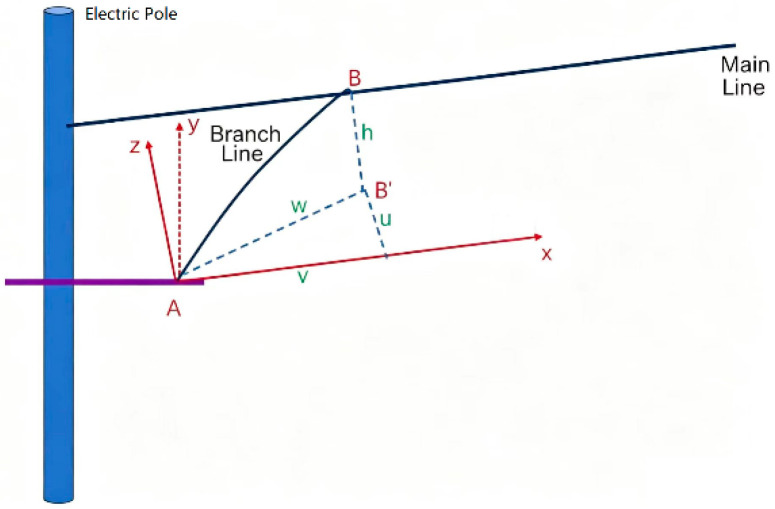
Relationship diagram of main lines, branch lines, and their connection nodes. Notes: *A* = origin; *x*, *y*, *z* = coordinate axes; vertical bar = electric pole; *AB* = branch line; top horizontal line = main line; *B*, *B*′, *w*, *h*, *u*, *v* denote geometric parameters.

Based on the above multi-modal perception processing pipeline, this section establishes a geometric workspace solving flow for insulated vehicle positioning, aiming to standardize the vehicle parking decision process and eliminate manual repeated trial adjustments during drainage wire clamping tasks. The core constraint condition is that dual manipulators need enough reachable workspace to finish wiring actions without triggering joint angle limits or colliding with adjacent charged lines. A parametric geometric solving framework is constructed to derive the optimal parking position, following the steps below (Figure 8):

1. Establish a local coordinate system taking the fixed connection point A of the drainage wire as the origin; the X-axis runs parallel to the main overhead line, the Z-axis is perpendicular to the main line, and the Y-axis conforms to the right-hand coordinate rule;

2. Input known structural parameters, including drainage wire total length, vertical height difference, and horizontal spacing between node A and the main line, fit the catenary parabolic sag curve, and solve all alternative candidate hanging points B;

3. Calculate the kinematic reachable workspace of dual manipulators corresponding to each candidate hanging point separately;

4. Extract the geometric center of each workspace as the preferred base position matching every hanging point;

5. Integrate all alternative positions to form an overall available workspace, and take its central coordinate as the final global parking position for the insulated vehicle.

The spatial coordinate calculation of hanging points depends on the classic parabolic sag mathematical model of flexible overhead conductors. The boundary condition at point A is substituted into the curve equation to solve the unknown coefficients, so as to calculate the spatial coordinates of all alternative operation points.

Traditional field operation totally relies on subjective judgment to adjust the vehicle position back and forth, leading to low preparation efficiency and hidden risks from human judgment errors. Different from the trial-and-error manual adjustment mode, this paper constructs a fixed geometric constraint-solving framework based on wire sag parametric modeling. It can output a determined parking coordinate through one round of calculation, standardizing the positioning procedure and cutting down on blind repeated vehicle movement in field tasks.

The candidate range for the hanging point is calculated as follows. (1) Fit the curve of the hanging line using the parabolic equation, determine the coordinates of the hanging point, and mark the hanging point as *B*. If the origin *A* is a point on the parabola, then *c* = 0, and the parabolic equation:

(1) $$y=ax^{2}+bx$$

The parabolic curve is shown as Equation (1), and its slope calculation formula is: d*y*/d*x* = 2*ax* + *b*; at the vertices of the function, the slope is 0, i.e., d*y*/d*x* = 0, so: 2*ax* + *b* = 0, 2*ax* = −*b*, *x* = −*b*/(2*a*). (2) The distance between the projection point *B*′ of hanging point *B* on the *x*-*y* plane and point *A* is denoted as *w*, and the coordinates of hanging point *B* are (*v*, *u*, *a* × *w*^2^ + *b* × *w*). From the geometric relationship, the coordinates of hanging point B are obtained as (*v*, *u*, *a* × (*v*^2^ + *u*^2^) + *b* × sqrt (*v*^2^ + *u*^2^), where *v* is the distance from *B*′ to the *y*-axis and *u* is the distance from *B*′ to the *x*-axis, then *w* = sqrt (*v*^2^ + *u*^2^). (3) According to the known parabolic arc length *s* between *A* and *B*, according to the definite integral arc length formula and their relationship:

(2) $$a=\left(h-b*w\right)/w^{2}$$

Arc length formula: $s=\int_{0}^{w}\sqrt{1+{\left(2a*x+b\right)}^{2}}dx$. The indefinite integration of the arc length formula yields:

(3) $$s=\frac{2a*x+b}{4a}\sqrt{1+{\left(2a*x+b\right)}^{2}}|\begin{array}{c} w\\ 0 \end{array}+\frac{1}{8a}\ln\frac{\sqrt{1+{\left(2a*x+b\right)}^{2}}+2a*x+b}{\sqrt{1+{\left(2a*x+b\right)}^{2}}-(2a*x+b)}|\begin{array}{c} w\\ 0 \end{array}$$

(4) $$s=\frac{1}{4(h-bw)}[\left(2h-w\right)\sqrt{w^{2}+{\left(2h-bw\right)}^{2}}-bw^{2}\sqrt{1+b^{2}}+w^{2}ln\frac{\sqrt{w^{2}+{\left(2h-bw\right)}^{2}}+2h-bw}{w(\sqrt{1+b^{2}}+b)}]$$

Substituting Equation (2) into Equation (3) yields Equation (4), where *b* and *w* are variables, and different values of b can lead to different values of *w*. Therefore, the set of hanging points {*B*_1_, *B*_2_, *B*_3_…, *B*_n_} is obtained. In step (2), the distance *D*(*B_i_*, *B_j_*) between any two candidates {*B_i_*, *B_j_*} is greater than 5 cm, and *D* is the Euclidean distance.

This paper adopts a dual-manipulator cooperative system to perform power operation tasks, including branch line mounting and wiring. Specifically, manipulator 1 undertakes wire grasping and insulation stripping, whereas manipulator 2 implements line hanging and wiring operations. According to the positional correlation of the two manipulators, the base coordinate origin of manipulator 1 is defined as *O*, and that of manipulator 2 is denoted as *O*′. Subsequently, distance constraint inequalities are formulated with reference to the link lengths of the manipulators: $\alpha_{A}<D\left(O,A\right)<\beta_{A}$, $\alpha_{B}<D\left(O^{\prime },B_{n}\right)<\beta_{B}$.

Establish distance constraints based on the length of the robotic arm (including the length of the insulating rod and tool): α_A_ = α_B_ = 0.9 m, β_A_ = β_B_ = 1.6 m; α_A_ and β_A_ respectively represent the minimum and maximum values of the position between the robotic arm and the root of the branch line, while α_B_ and β_B_ respectively represent the minimum and maximum values of the position between the robotic arm and the hanging point.

The set of robot positions that satisfy the condition for any hanging point *Bn* is {$O_{1}^{n},O_{2}^{n},O_{3}^{n},\ldots ,O_{m}^{n}$}, which is the workspace corresponding to the hanging point *B_n_*. The distance D ($O_{i}^{n},O_{j}^{n}$) between any two {$O_{i}^{n},O_{j}^{n}$} is greater than 5 cm. Then, the center of the set {$O_{1}^{n},O_{2}^{n},O_{3}^{n},\ldots ,O_{m}^{n}$} is taken as the optimal working position for the hanging point *B_n_*:

(5) $$O_{mn}^{n}=\frac{O_{1}^{n}+O_{2}^{n}+O_{3}^{n}+\cdots O_{m}^{n}}{n}$$

Furthermore, for all B_n_, the set of optimal operating positions for all hanging points is denoted as {$O_{m1}^{1},O_{m2}^{2},O_{m3}^{3},\ldots ,O_{mn}^{n}$}, which forms a new workspace for the robotic arm. The center of this workspace is taken as the optimal operating position for the robotic arm:

(6) $$O_{e}^{all}=\frac{O_{m1}^{1}+O_{m2}^{2}+O_{m3}^{3}+\cdots O_{mn}^{n}}{n}$$

#### 3.2.3. Calculation of Grabbing and Stripping Positions for Branch Lines

Accurate extraction of wire feature points is a prerequisite for automated live-line manipulation. Conventional positioning algorithms require manual selection of main and branch wire endpoints on LiDAR point clouds before calculating stripping and grasping positions. This manual operation imposes strict requirements on operator proficiency, reduces overall task efficiency, and introduces inconsistent positioning errors from human intervention.

To overcome this limitation, this paper proposes an automatic wire identification scheme combining reflective markers and LiDAR point cloud processing, which eliminates manual point selection and realizes autonomous solving of grasping and stripping coordinates. The implementation pipeline of the proposed method is summarized as follows:

1. Two reflective markers are pasted on the target branch wire; the on-board LiDAR scans and stitches point cloud data of the operation area.

2. Range filtering and highlight threshold segmentation are executed on the stitched point cloud to extract point clusters corresponding to reflective markers.

3. Statistical filtering and cluster analysis are adopted to calculate the centroid of each marker cluster as effective feature coordinates.

4. Euclidean distances between marker centroids are solved. Combined with the fixed spacing feature of markers on a single wire, the system matches each group of markers to the corresponding overhead conductor and completes automatic wire segmentation.

Compared with manual point selection adopted in existing live-working robots, the marker-assisted LiDAR identification scheme avoids subjective human error, standardizes feature extraction procedures, and accelerates the calculation of stripping and grasping positions in actual field operations.

### 3.3. Collaboration Mechanism of Dual-Arm Robots

**Figure 9:**
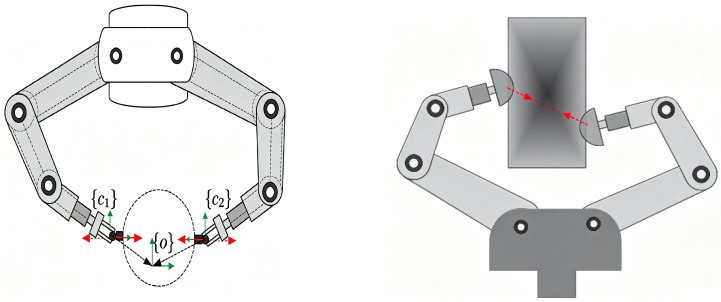
Two grasping methods of dual-arm robots. Red arrows represent force transmission directions; green arrows indicate the positive directions of coordinate axes; {*c*_1_}, {*c*_2_} and {*o*} denote manipulator and workpiece coordinate systems, respectively. The left subplot shows rigid fixed grasping; the right subplot presents friction contact grasping.

Dual-arm coordinated manipulation forms a closed kinematic chain when two end-effectors jointly clamp targets, which fundamentally differs from single-arm open-chain modeling. Two typical coordinated grasping modes are analyzed in Figure 9.

1. Rigid Fixed Grasping (Left Subgraph of Figure 9):

Two manipulators form a rigid constraint connection with the workpiece without relative sliding between the end-effector and target. Arm motion is transmitted to the object via bilateral geometric constraints, suitable for heavy or large-diameter live-line fittings that cannot be stably supported by a single manipulator.

2. Friction Contact Grasping (Right Subgraph of Figure 9):

Objects are held only by contact friction force, with unilateral force constraints applied by robotic arms. This mode matches human dexterous finger grasping and relies heavily on contact friction modeling.

The live-line wire clamping task in this paper mainly adopts rigid fixed grasping, so this work focuses on the corresponding quasi-static modeling method. A grasp matrix is constructed to map velocity and generalized force between the grasping point coordinate frames and the object centroid frame:

(7) $$G=\left(\begin{array}{cccc} I_{3} & O_{3} & I_{3} & O_{3}\\ -S(p_{oc1}) & I_{3} & -S(p_{oc2}) & I_{3} \end{array}\right)$$

where $p_{oci}$ denotes the position vector from frame *c*_1_ to object centroid *O*; S(·) represents the skew-symmetric matrix for the cross product operation; and $I_{3}$ and $O_{3}$ are 3-order identity and zero matrices, respectively. A virtual rigid link can be defined between each end-effector grasping point and the workpiece centroid for kinematic mapping.

Coordinated task space equations take absolute and relative displacements of dual arms as state variables, supporting modeling regardless of physical contact state. Accurate contact friction modeling is essential for friction grasping derivation, which belongs to an independent research field and is not involved in our live-line task scenario; thus, related theoretical derivation is omitted here [14,15].

## 4. Experiments and Result Analysis

### 4.1. Experimental Platform

**Figure 10:**
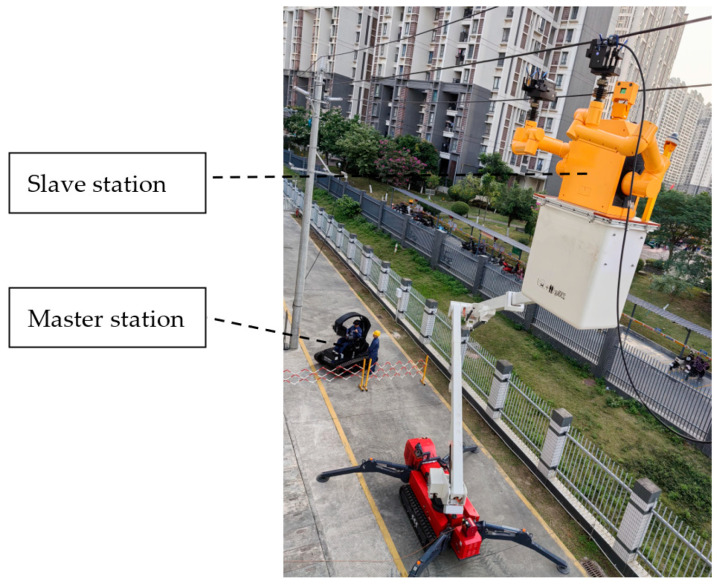
Prototype of the live working robot system.

The proposed master–slave robotic platform is built to validate the four-dimensional safety supervision framework and MR force-feedback teleoperation strategy developed in this paper. The master control terminal adopts a flexible tracked chassis integrated with a main control cabinet and twin master manipulators for human-in-the-loop manipulation. The slave execution unit is installed on a crawler insulated spider aerial platform, carrying dual insulated manipulators and supporting peripheral functional modules including central controllers, visual recognition units, pose guidance subsystems, real-time monitoring equipment, power supply modules, and miniature meteorological sensors. The complete physical prototype is displayed in Figure 10.

This prototype is customized for 10 kV overhead distribution maintenance scenarios in South China. All trials were implemented at a standardized professional training base in Huangpu District, Guangzhou, equipped with horizontal and triangular overhead line test racks; all test data adopted in this paper were acquired from triangular line groups. Due to industry qualification restrictions for high-voltage live tasks, only two fully certified professional operators with systematic pre-training participated in all comparative experiments. After three rounds of internal debugging and official third-party performance certification, the prototype meets all preset technical indicators and gains approval for on-site live-line demonstration and further practical verification on real power grid lines.

### 4.2. Experimental Results

**Figure 11:**
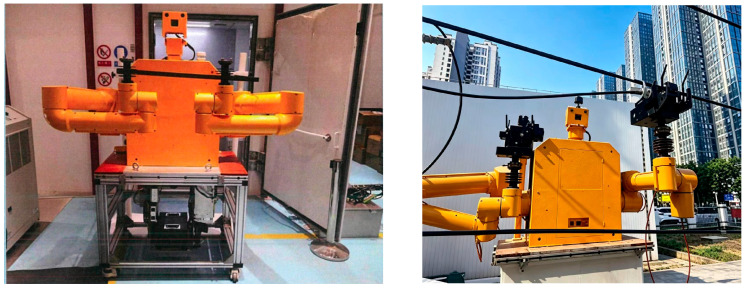
Third-party testing report and photos of the testing process. Laboratory test platform (**left**) and field operation photos of the robotic equipment (**right**).

After prototype fabrication, a nationally accredited third-party testing institute in P.R. China was commissioned to perform comprehensive inspections against the proposed design specifications and four-quadrant safety constraints. According to the official test report, the developed prototype can satisfy the designed functional, performance, and safety technical indicators under standard laboratory test conditions. Partial photographs of key indicator testing procedures and corresponding test outcomes are presented in Figure 11.

To verify the insulation safety performance of the slave station, AC withstand voltage tests were implemented in a certified laboratory following standards, including GB/T 17626.2 [20] and third-party test specifications. During the test, an AC voltage of 45 kV was continuously applied for 1 min. No breakdown, flashover, or abnormal overheating was observed. Under the 45 kV AC excitation, the measured leakage current was controlled below 100 μA. Three identical prototype samples were tested for repeatability, and all samples yielded qualified results with low data dispersion, which demonstrates acceptable repeatability of the insulation test. Within the given laboratory test conditions, the insulation design matches the required safety criteria for 10 kV live-line tasks.

In order to prove electromagnetic communication safety, according to the testing conducted in the electromagnetic compatibility laboratory and the issued testing report, four testing contents, including electrostatic discharge immunity testing, power frequency magnetic field immunity testing, pulse magnetic field immunity testing, and damping oscillation magnetic field immunity testing, were carried out in accordance with the testing criteria such as GB/T17626.2 [20]. The testing results showed that the robot maintained normal function and continuous master–slave communication without interruption during the whole testing process. Multiple repeated measurements were performed for each immunity item, and all batches of experimental observations obtained relatively stable response performances without severe abnormal fluctuations. The EMC design presents qualified performance under standard laboratory interference tests.

Transmission latency indicators of the wireless communication system were fully tested and recorded by an accredited third-party testing institution under typical 10 kV tower electromagnetic interference conditions. Under the standard test environment specified in the inspection scheme, the average video transmission latency was measured as 98 ms, while the average delay of independent-band control signals reached only 12 ms. The separation of control signal and video stream frequency bands helps avoid wireless bandwidth congestion and mitigate delay fluctuation caused by power-frequency harmonic interference around overhead lines. From an engineering application perspective, the measured delay level supports a timely master–slave linkage response in the given test environment, and meets the basic real-time requirement to lower the risk of lag-induced collision during teleoperation. Due to the on-site rapid inspection procedure of the third-party laboratory, complete continuous multi-cycle sampling raw data were not archived, but the test environment strictly complied with the power industry on-site detection standards, and the measured index values can reflect the basic communication performance of the system under calibrated test conditions.

According to the testing report, the embedded monitoring configuration provides auxiliary conditions to guarantee process safety during operation. Firstly, the robot is equipped with meteorological and temperature sensors to monitor ambient wind speed and temperature, which can provide reference data for adjusting operation strategies. Secondly, the matched UAV device serves as an auxiliary monitoring unit to observe the operation scene from high angles for risk warning, while recording footage for subsequent work review. Combined with the above third-party detection items and field trials, the designed system possesses the auxiliary safeguard structure corresponding to the four-dimensional safety framework.

**Figure 12:**
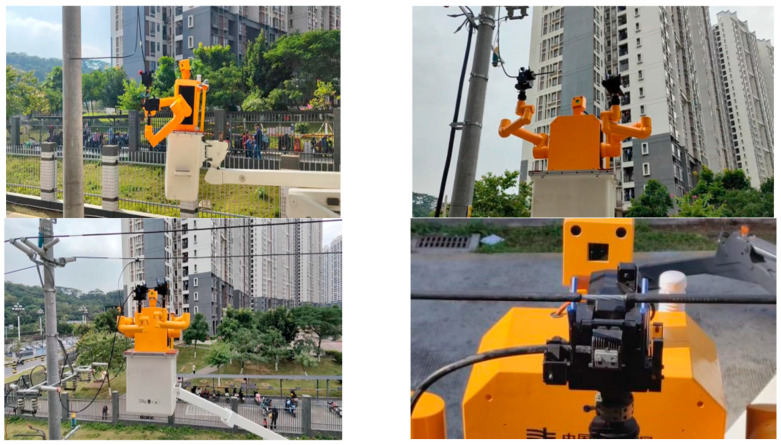
Working process display.

Practical experiments carried out by qualified operators at the professional training field are summarized in Figure 12. Within the scope of our comparative trials, combining visual-force auxiliary display with conventional force feedback shows favorable task completion outcomes for wire clamping and wire stripping positioning operations. We recruited two novice trainees with basic theoretical knowledge for comparative trials. After brief standardized training, tasks relying merely on physical force feedback presented relatively high failure probability, ranging from 50% to 60% across 20 repeated trials. By introducing visual auxiliary force display together with physical force feedback, the success count rises to 18 out of 20 trials in this test group, presenting a preferable task completion rate under the same test setup.

The end-effector positioning accuracy of the robot was verified through a standardized test organized by a third-party testing agency. Static test conditions with stable illumination and weak electromagnetic interference were adopted as the baseline working environment. A laser-calibrated manipulator with a benchmark absolute positioning accuracy of 0.52 mm was used as the reference calibration equipment. The test flow covered eight representative sampling points distributed in the full working space: the robotic end effector was manually positioned to each calibration marker, then moved over a long distance, and returned to the target point under visual fusion positioning guidance. The third-party report confirmed that the maximum absolute positioning error under standard test conditions is 4.4 mm. Minor positioning deviation mainly originates from three practical engineering factors: subtle light intensity changes, tiny mechanical joint clearances of the insulating arm, and partial occlusion of wire fittings to visual feature points. The measured maximum error is smaller than the routine allowable error limit (±10 mm) for 10 kV live maintenance work, meaning the positioning scheme meets the precision threshold required in standard testing scenarios. No safety accidents occurred during the whole detection process. It should be noted that the third-party inspection focused on obtaining characteristic limit indicators rather than storing massive continuous measurement raw data, so full sets of repeated test records are not available, but the detection process strictly follows unified power industry test specifications.

In the “10 kV Distribution Network Non-Power Outage Operation Specification (Trial)” and related practical reports, the median average manual operation time of live T-connection of three-phase drainage lines across diverse field scenarios is approximately 2.25 h (from 1.5 to 3 h) [25]. This statistical benchmark covers varying operation complexities, operator proficiency levels, and site environmental conditions from public industry records. In controlled training ground tests with unified preparation conditions, the average working duration of the robotic system is around 1.74 h. The calculated time-saving proportion is merely a rough reference value, because the industry public statistical data and our controlled experiment adopt inconsistent test boundaries and pre-work preparation standards. Hence, this proportion cannot be treated as rigorous quantitative evidence. Only within our fixed test setup can we observe a moderate reduction in operating time compared with cited manual statistical data.

To preliminarily verify the effectiveness of the insulation bucket positioning function, comparative experiments were conducted with two certified professional operators (A and B). Tasks belonging to 10 kV live-line work belong to high-risk special operations, which require operators to hold official industry qualification certificates for safety compliance. Fully certified practitioners available for long-duration repeated field trials are very scarce, so only two experienced, qualified operators are enrolled to finish all groups of contrast tests in this paper. Each operator completed two test conditions: trials with the insulation bucket positioning function enabled (A1, B1), and trials with this positioning function disabled (A2, B2). All repeated tests from the two operators are collected for internal comparison, while relevant statistical conclusions are only constrained to this small sample group, without generalization to operator groups with different proficiency or external complex field environments. The four subgraphs of Figure 12 illustrate the sequential procedures of grasping, threading, and cable connection.

**Figure 13:**
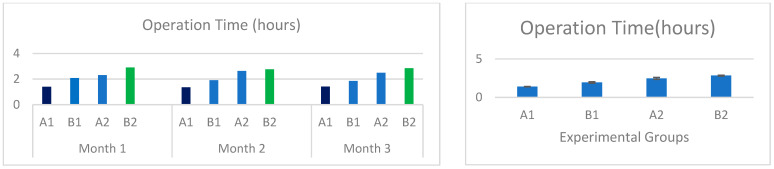
Comparison of average operation time under four experimental schemes after three rounds of periodic tests.

In the robot system working duration tests, we observed a general downward trend in average operating time alongside growing operational proficiency, while obvious inter-individual performance differences were also identified within our limited participant pool. In third-party verification tests, each operator performed repeated trials, yielding an overall average operating time of approximately 1.74 h. Subgroup results showed an average of 2.08 h for case A1 and 1.4 h for case B1. In the two-month follow-up comparative test, Operator A showed only a marginal improvement in performance, while Operator B significantly reduced operation time but still fell short of Operator A’s efficiency level, as shown in the left subgraph of Figure 13. These observations indicate that the operator’s personal operating habits and proficiency produce obvious impacts on task efficiency in our tested sample group. The core design emphasis of this framework focuses on multi-layer safety protection rather than efficiency promotion, and further economic benefit analysis will be explored in future follow-up research.

The subgraph on the left of Figure 13 compares the time performance of Operator A and Operator B over three consecutive months. When the insulation bucket positioning function was disabled, a measurable prolongation in operating duration was observed for both participants. The combined average time of A2 and B2 reached roughly 2.67 h, which was even inferior to the manual operation efficiency. Paired statistical comparison shows that the time difference after shutting down the positioning function is not a random fluctuation under our test conditions. Within the limited qualified sample group in this paper, the one-time positioning function helps cut down blind vehicle adjustment steps and brings favorable practical value for the tested fixed operation scenario.

Three batches of periodic field experiments were implemented for working groups A1, A2 (robot operation), and B1, B2 (manual operation), respectively, under identical environmental conditions, preparatory work scope, and operational procedures. The subgraph on the right of Figure 13 compares the averaged time consumption of four schemes, with standard deviation marked by error bars to evaluate data stability. Solid bars in this Figure denote the average operation time of repeated experiments, while error bars represent standard deviation, which quantitatively describes data fluctuation and experimental reproducibility. Under the unified test environment, the robotic group consumes less time on average, and small standard deviations reflect stable repeated test results for our fixed experiment setup.

Operators are categorized into novice and skilled groups according to accumulated training hours. Combined with experimental data illustrated in Figure 13, operation time and data dispersion under different proficiency levels are compared numerically. It can be found that insufficient operational experience tends to lengthen working duration and increase data fluctuation, while skilled operators maintain relatively stable manipulation efficiency in our test environment.

### 4.3. Field Application Verification

**Figure 14:**
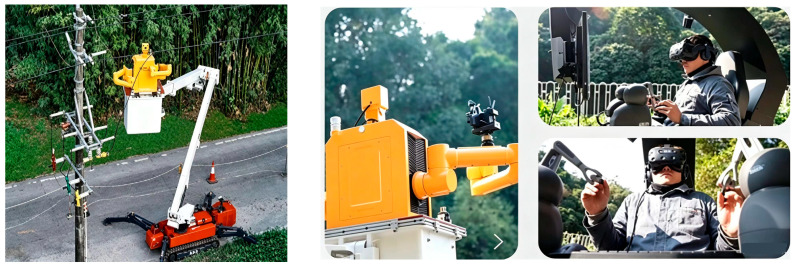
On-site operation of the MR teleoperated live-line working robot.

The self-developed “Wukong” MR teleoperation robot completed its first on-site wire connection trial at Guangzhou Science City on 21 December 2023, as shown in Figure 14. This paper presents a practical engineering case of MR teleoperation deployed for overhead 10 kV distribution maintenance under four-dimensional safety constraints. A second field verification was conducted on 31 October 2025 for 10 m high-altitude fault handling. Dual-insulated manipulators support stable tool rotation, with operators completing remote control via MR head-mounted displays inside ground master cabins. All field trial performance data are archived in official industry technical reports.

To further interpret the source of system stability and task efficiency under the tested scenarios, supplementary mechanism analysis is provided below. The overall safety margin does not merely rely on the passive insulating mechanical components of the manipulator. Instead, the constructed four-dimensional safety framework establishes layered risk interception logic. The insulation dimension provides fundamental high-voltage breakdown prevention; periodic handshake detection cuts off actuator movements once master–slave communication loses synchronization; human–robot interaction adds trajectory pre-judgment collision checking before control commands are transmitted; and drone auxiliary aerial monitoring fills blind observation areas during high-altitude operation. The two-tier mechanism, including early warning reminder and motion locking, forms progressive risk isolation, which expands the overall safety boundary under given test environments.

In terms of working efficiency optimization, the complete multi-sensor calibration procedure, together with catenary geometric workspace solving, cancels repeated blind parking adjustments relying entirely on operator subjective judgment in traditional operation modes. In addition, the end-effector-following force display design inside the MR view avoids frequent sight switching between fixed force indicator panels and operating targets, lowering the probability of misoperation caused by visual distraction. These customized procedural and structural designs simplify pre-operation preparation steps and maintain a relatively steady manipulation rhythm in our comparative tests.

Referring to domestic and overseas representative prototypes and commercial equipment summarized in Table 1 of the introduction section, most existing studies concentrate on manipulator mechanical structure optimization, positioning algorithm precision promotion, or single-module communication anti-interference capability. Their safety measures mostly adopt only passive insulating materials, matched with simple single-condition shutdown protection after signal disconnection, without a systematic multi-index whole-process risk supervision framework. Traditional teleoperation modes depend on fixed-screen video monitoring, lacking intuitive real-time force presentation feedback for contact status.

Distinguished from the above schemes, this paper builds a full-cycle four-dimensional monitoring and protection system, and adopts the follow-up embedded force visualization mode adapted for mixed reality teleoperation. Meanwhile, the formulated geometric constraint positioning workflow reduces redundant trial-and-error vehicle debugging work. Nevertheless, this research still carries certain restrictions based on current test conditions.

## 5. Limitations and Future Research Directions

### 5.1. Current Research Limitations

This work presents a multi-sensor fusion perception and human-in-the-loop teleoperation system for 10 kV live-line maintenance robots, and the feasibility, safety, and operational performance of the developed prototype are validated through laboratory tests and field trials. Nevertheless, the current study still contains several inherent limitations under complex practical engineering scenarios, which are objectively analyzed in this section to provide clear guidance for subsequent optimization and in-depth research.

First, limited scenario and task adaptability. All experimental verifications in this study are conducted under benign meteorological conditions and weak electromagnetic interference environments, with experimental tasks mainly focusing on conventional drainage wire connection operations. Extreme working scenarios, including strong wind vibration, heavy rainfall, and intensive on-site electromagnetic coupling, are not fully explored. Meanwhile, typical complex live-line tasks such as insulator replacement and foreign object removal are excluded from the test dataset. Therefore, the universality and generalization ability of the proposed system in diverse and harsh working conditions need further verification.

Second, insufficient universality of experimental conclusions. Restricted by the scarcity of professionally certified live-line maintenance operators, only two skilled technicians participated in the comparative human–machine operation tests in this work. The obtained results regarding operational efficiency and human–machine adaptation characteristics are only applicable to this small sample. The performance differences among operators with different proficiency levels and operational habits are not systematically discussed, which limits the statistical persuasiveness and popularization of the experimental conclusions.

Third, simplified theoretical assumptions of the positioning algorithm. The catenary geometric positioning model adopted in this study is established based on an ideal overhead conductor state. In actual complex field environments, unpredictable interference factors such as violent wire vibration, surface dust accumulation, and partial target occlusion will degrade the accuracy of point cloud segmentation and spatial positioning. In addition, the current system lacks adaptive parameter adjustment and anti-interference optimization modules for variable working conditions, resulting in limited environmental robustness of the perception and positioning subsystem.

Fourth, lack of long-term operational stability verification. All field tests conducted in this study are short-term intermittent demonstration experiments. Long-duration continuous operation endurance tests under full working conditions have not been completed. The long-term working stability and durability of key components, including wireless communication modules and layered insulation structures, cannot be fully verified. The aging performance and continuous operational reliability of the prototype in long-term field service still require further systematic evaluation.

### 5.2. Future Research Prospects

Aiming at the aforementioned limitations, targeted improvement schemes and future research directions are proposed to further enhance the environmental adaptability, operational robustness, and engineering practicability of the live-line working robot system.

(1) Enrich experimental scenarios and sample diversity. Subsequent research will supplement systematic experiments under extreme meteorological conditions and strong electromagnetic interference scenarios, and expand the task library to cover insulator replacement, foreign object removal, and other mainstream live-line operations. Meanwhile, more operators with different skill levels will be recruited to participate in comparative tests, so as to verify the system’s adaptability to different users and improve the universality of research conclusions.

(2) Optimize the robustness of multi-modal perception algorithms. To address the positioning deviation caused by wire vibration and target occlusion, dynamic conductor sag correction and adaptive point cloud filtering modules will be developed. By introducing real-time environmental interference feedback, the system’s anti-interference ability and positioning accuracy in complex field scenarios can be effectively improved.

(3) Realize dynamic optimization of the multi-dimensional safety framework. The existing fixed early-warning and locking thresholds will be upgraded to adaptive dynamic thresholds. Combined with real-time environmental parameters such as electromagnetic intensity and ambient humidity, the system can adjust safety constraint standards intelligently, further optimizing the layered risk interception capability of the four-dimensional safety guarantee system.

(4) Carry out lightweight design and engineering economic evaluation. Aiming at the problem of insufficient trafficability in narrow distribution operation corridors, the mechanical structure of the prototype will be lightweight and optimized. Furthermore, quantitative cost–benefit analysis will be conducted to evaluate the engineering application value of the system, providing practical references for large-scale popularization and deployment.

(5) Upgrade autonomous operation capabilities from semi-auxiliary to adaptive full-autonomous control. The current prototype realizes basic semi-autonomous auxiliary planning under manually defined task boundaries. Future work will further embed intelligent decision-making and adaptive task scheduling modules to support autonomous operation in unstructured and dynamically changing scenarios. By integrating real-time environmental perception results and safety constraint thresholds, the robot can independently complete target recognition, obstacle avoidance, and continuous operation without frequent human intervention, thereby improving the overall intelligence level and field adaptability of the live-line maintenance system.

## Figures and Tables

**Table 1:** Comparison between reported robotic schemes and the proposed system.

	Reported Traditional Robotic Schemes	Proposed Scheme in This Work
**Environmental perception**	Single LiDAR or monocular vision	Binocular-LiDAR multi-frame fusion, customized reconstruction for vertically distributed pole fittings
**Operation execution mode**	Basic dual-arm master-slave manipulation	Force-feedback MR master-slave coordinated control for double-insulated manipulators
**Decision framework**	Operator visual monitoring + simple computer planning	Human-in-the-loop operation mode matched with layered safety assessment architecture
**Overall operational efficiency**	Repeated manual vehicle adjustment extends preparation time	Continuous data collection during lifting simplifies pre-operation positioning steps
**Safety guarantee**	Single mechanical insulation + basic electrical protection	Four-dimensional hierarchical safety assessment covering insulation, EMC, human–machine interaction and full-process supervision

## Data Availability

The raw experimental data generated in this study are available from the corresponding author upon reasonable request.
